# Global Cortical Thinning Predicts Slower Forward and Backward Walking in Multiple Sclerosis

**DOI:** 10.1111/ejn.70412

**Published:** 2026-01-21

**Authors:** A. S. Monaghan, P. G. Monaghan, T. N. Takla, N. E. Fritz

**Affiliations:** ^1^ School of Psychology Queen's University Belfast UK; ^2^ Department of Health Care Sciences Wayne State University Detroit Michigan USA; ^3^ Translational Neuroscience Program Wayne State University Detroit Michigan USA; ^4^ Department of Neurology Wayne State University Detroit Michigan USA

**Keywords:** cerebral cortex, cortical thinning, fall risk, gait, gray matter, multiple sclerosis, walking

## Abstract

Gait impairment and falls are common in multiple sclerosis (MS), yet the neural substrates contributing to mobility decline remain poorly understood. While prior studies have linked regional gray matter atrophy to motor outcomes, the role of diffuse cortical changes in complex gait tasks, such as backward walking, is less clear. This study examined whether diffuse cortical thinning is associated with forward and backward walking speed, and whether these relationships differ between fallers and nonfallers in MS. Forty‐three individuals with MS (55 ± 10 years; 65% female) completed forward and backward Timed 25‐Foot Walk assessments and high‐resolution structural MRI. Cortical thickness and gray matter volume were estimated. Region‐specific associations with gait speed were examined using regression with false discovery rate (FDR) correction. Principal component analysis revealed a global cortical thinning component that was associated with slower forward (*β* = −0.065, adjusted *R* ^2^ = 0.14) and backward (*β* = −0.061, adjusted *R* ^2^ = 0.19) walking speeds. In contrast, volumetric components did not significantly predict gait. Fallers and nonfallers did not differ in gait speed or cortical thinning, but exploratory moderation suggested stronger cortical thinning–gait associations in fallers, although effects did not remain significant after FDR correction. These preliminary findings suggest that diffuse cortical thinning is a consistent neural correlate of gait slowing in MS, with exploratory evidence suggesting that this relationship may be stronger among individuals with recent falls.

## Graphical abstract

In 43 people with MS, we measured forward/backward walking speeds and examined cortical thickness and volumes across predefined ROIs, then reduced features with PCA. Regional effects were inconsistent, but the first PCA component, a global cortical‐thinning signature, was associated with slower forward and backward walking. The thinning‐speed relationship appeared stronger in recent fallers.

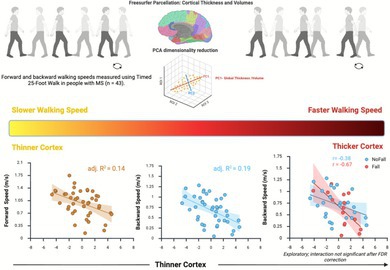

**Abbreviations**
ANCOVA: analysis of covariance
BWK: backward walking
CI: confidence interval
CV: cross‐validation
FDR: false discovery rate
FWD: forward walking
GM: gray matter
ICV: intracranial volume
MEMPRAGE: multi‐echo magnetization‐prepared rapid gradient echo
MRI: magnetic resonance imaging
MS: multiple sclerosis
PC: principal component
PCA: principal component analysis
PDDS: Patient Determined Disease Steps
RMSE: root mean square error
ROI: region of interest
SE: standard error
T25FW: Timed 25‐Foot Walk
ThickPC1: first thickness principal component
ThickPC2: second thickness principal component
VolPC1: first volume principal component
VolPC2: second volume principal component

## 1 Introduction

Multiple sclerosis (MS) is a chronic neurological disease characterized by demyelination and neurodegeneration, leading to progressive motor impairments. Gait and balance difficulties often emerge early, even among individuals with minimal disability, and typically worsen as the disease advances (Cattaneo et al. 2002; Alharthi and Almurdi 2023). These mobility impairments contribute to a high risk of falls, with up to 70% of people with MS experiencing at least one fall within a six‐month period (Finlayson et al. 2014; Matsuda et al. 2012). Falls are associated with injury, reduced independence, and diminished quality of life, motivating efforts to identify neural correlates of mobility decline and fall risk (Coote et al. 2020; Finlayson et al. 2006). Despite this high prevalence, the neural substrates that contribute to fall risk in MS remain poorly understood. Traditional gait assessments in MS predominantly focus on forward walking, but emerging evidence highlights the value of more complex locomotor tasks, such as backward walking, for detecting subtle mobility impairments and fall risk (Monaghan, Takla, et al. 2024; Seferoğlu et al. 2025). Backward walking imposes greater demands on postural control, neuromuscular coordination, and proprioceptive feedback, engaging multiple neurophysiological systems affected in MS (VanNostrand et al. 2024; Hackney and Earhart 2009; DelMastro et al. 2023). Prior work has shown that backward walking is a strong predictor of fall risk in MS, outperforming forward walking in distinguishing fallers from nonfallers (Edwards et al. 2020). Thus, incorporating both forward and backward gait assessments may provide a more comprehensive approach to monitoring mobility decline and fall risk in MS.

Neuroimaging studies in MS demonstrate that both cortical and subcortical gray matter atrophy are associated with impaired motor function, including gait and turning performance, and sensory function. Thinning of the motor cortex, particularly in the precentral and paracentral gyri, has been linked to reduced dynamic movement abilities and greater disability (Swanson and Fling 2023; Sailer et al. 2003), while subcortical volume loss, including the thalamus and basal ganglia, also contributes to mobility decline (Pareto et al. 2015). Thinning of the anterior cingulate, parietal operculum, and inferior frontal gyrus has been linked with poorer vibration perception, particularly in progressive forms of MS (Fritz et al. 2019). Both regional and global cortical thinning appear relevant: region‐specific atrophy in sensorimotor areas shows distinct associations with disability, while diffuse cortical thinning also emerges as the disease progresses (Swanson and Fling 2023; Sailer et al. 2003; Pareto et al. 2015; Geisseler et al. 2016). Additionally, the integrity of white matter tracts, such as the corticospinal tract, is closely related to motor cortex thinning and disability, underscoring the joint influence of gray and white matter on motor outcomes (Bergsland et al. 2015). Yet most studies have focused on forward walking or general motor function, with limited investigation of the neural correlates of more complex tasks such as backward walking. Moreover, whether fall history moderates the relationship between gray matter morphometry and gait performance remains largely unexplored, representing a critical gap in our understanding of how structural brain changes contribute to real‐world fall risk in MS.

This study's primary aim was to test whether cortical thinning and gray‐matter volume patterns are associated with forward and backward walking speed in MS. As a secondary, exploratory aim, we tested whether prospective fall status moderates the association between global cortical thinning and walking speed. We hypothesized that diffuse cortical thinning would be associated with slower walking speeds and that these effects would be amplified in individuals with a history of falls. This study aims to clarify the structural underpinnings of complex gait impairment and falls in MS. Identifying morphometric markers of gait and fall risk has the potential to improve risk stratification and inform rehabilitation strategies targeting fall prevention.

## 2 Methods

### 2.1 Participants

All study procedures were approved by the Wayne State University (WSU) Institutional Review Board, and all participants provided written informed consent in accordance with the Declaration of Helsinki. Individuals were eligible for participation if they: (1) had a neurologist‐confirmed diagnosis of MS; (2) were between the ages of 18 and 65 years; (3) had the ability to ambulate independently or with an assistive device; (4) had normal or corrected‐to‐normal vision; and (5) were able to follow study instructions. Exclusion criteria included: (1) MS relapse in the previous 8 weeks; (2) presence of an acute orthopedic condition affecting gait; (3) diagnosis of a comorbid neurological condition; (4) use of corticosteroids in the past 30 days; (5) current use of dalfampridine or 4‐aminopyridine; (6) any MRI contraindications (e.g., metallic implants); or (7) self‐reported pregnancy.

### 2.2 Study Protocol

All procedures were completed during a single study visit. Participants first completed a series of self‐report questionnaires, including demographic information (age, sex, and race/ethnicity), disease history (symptom duration and MS subtype), and disability status (Patient Determined Disease Steps, PDDS). MRI scans and clinical gait assessments were completed during a single study visit. Depending on scanner availability, MRI acquisition occurred either immediately before or after clinical testing; however, for most participants, the interval between MRI and gait assessments was within approximately 30 min. Following the visit, participants received weekly fall surveys for 6 months, in which they reported the number of falls experienced over the prior week.

### 2.3 Forward and Backward Walking Assessments

Participants completed the Timed 25‐Foot Walk (T25FW) both forward and backward at their typical walking speed. Participants completed two trials in each direction, and the completion times were averaged. To derive walking velocity, the 25‐ft distance was divided by the mean time for forward and backward walking, yielding two distinct velocity values for analysis. The T25FW is a widely applied clinical measure in MS populations and demonstrates strong validity and reliability (Monaghan, Takla, et al. 2024; Motl et al. 2017).

### 2.4 MRI Data Acquisition, Processing, and Quality Control.

Structural MRI data were acquired on a 3 T Siemens MAGNETOM Verio scanner using a 32‐channel head coil. High‐resolution T1‐weighted images were collected with a multi‐echo magnetization prepared rapid gradient echo (MEMPRAGE) sequence (176 slices, 1 mm isotropic resolution, field of view = 256 mm, GRAPPA = 2, flip angle = 7°, TR = 2530 ms, TEs = 1.79/3.65/5.51/7.37 ms, TA = 6:55).

Images were processed in FreeSurfer v7.2 using the automated cortical surface reconstruction and parcellation pipeline. Based on neuroimaging studies in MS associating cortical and subcortical gray matter atrophy with impaired motor function (Swanson and Fling 2023; Sailer et al. 2003; Pareto et al. 2015), regional measures of cortical thickness (mm) and volume (mm^3^) were extracted from the Desikan–Killiany parcellation (aparc) for the predefined regions of interest described in the Results (e.g., inferior parietal, supramarginal, subcortical gray matter, precentral, postcentral, precuneus, superior frontal, caudal middle frontal). Because of the small sample size and significant inter‐regional collinearity, subcortical gray matter was treated as a combined volume rather than decomposed into individual structures to reduce multiple comparisons and improve statistical stability. Left‐ and right‐hemisphere values were averaged to yield a single mean value for each region per participant.

All scans underwent visual quality control for motion artifacts and segmentation accuracy. Cases with major segmentation errors were excluded from further analysis. To account for head size, cortical volume measures were adjusted for intracranial volume (ICV) using an ANCOVA approach, and all subsequent analyses used ICV‐adjusted bilateral volumes.

### 2.5 Statistical Analyses

All analyses were conducted in R 4.3.1. Continuous variables were first inspected for normality via Shapiro–Wilk tests (all *p* > 0.10). Prospective six‐month fall status was coded as a binary factor after excluding one subject with missing falls data (*n* = 42 for all subsequent analyses). Individuals who reported at least two falls in the six‐month period were classified as fallers, whereas individuals who reported < 2 falls were classified as nonfallers.

Separate linear regressions were used to predict each gait measure from each of our seven thickness regions and eight ICV–adjusted volumetric ROIs. For volume models, we included the estimated total ICV as a covariate. Model diagnostics included variance inflation factors (flag > 5), Cook's *D* (> 4/*n*), and leverage (> 2 × mean). The Benjamini–Hochberg false discovery rate correction was applied within each ROI set (thickness models: *q* across 7 tests; volume models: *q* across 9 tests). Pearson correlations and scatterplots assessed and visualized the associations between cortical structures and forward and backward walking speed, which were trending in the regression models.

Principal component analysis was conducted on the *z*‐score thickness and volume data to reduce dimensionality and capture patterns of atrophy. A combination of scree‐plot inspection and variance explained supported the retention of principal components (PCs). Individual PC scores were entered simultaneously into linear models predicting forward and backward walking speed. Model performance was summarized by adjusted *R* ^2^, and 10‐fold cross‐validation yielded out‐of‐sample RMSE and CV *R* ^2^. For study‐planning purposes, effect‐size‐based sample size estimates were derived from the primary regression models and are reported in Supporting Information.

To characterize group differences, PCs and forward and backward walking speed were compared between fallers and nonfallers using Welch's *t*‐tests, reporting Cohen's *d* effect sizes. The interaction between fall history and PCs was assessed to determine if fall history moderated the relationship between gray matter atrophy and walking speed. The four raw interaction *p*‐values were adjusted for FDR, and each interaction coefficient was bootstrapped (1000 resamples, percentile CIs) to identify robust effects whose 95% intervals excluded zero. Interaction plots were generated, and Pearson's *r* and *p*‐values were calculated for associations within fallers vs. nonfallers. All inferential tests were two‐tailed (*α* = 0.05), with both raw and FDR‐corrected *q*‐values reported.

## 3 Results

### 3.1 Descriptive Statistics

Participant characteristics and primary gait measures are summarized in Table 1. Of 43 persons with MS (mean ± SD age = 47.77 ± 9.98 years; 79% female; disease duration = 15.26 ± 9.64 years), forward walking speed averaged 0.98 ± 0.36 m/s and backward speed 0.69 ± 0.33 m/s. All gait variables were approximately normally distributed. One subject with missing prospective falls data was excluded from analyses involving fall status (*n* = 42 for Sections 3.6.1–3.6.2). Of the remaining participants, 27 (64.3%) were classified as nonfallers and 15 (35.7%) as fallers based on six‐month prospective fall monitoring. Among fallers, the median number of falls over 6 months was 5 (range: 0–60). When stratified by fall status (Table 1), fallers and nonfallers did not differ significantly in age, height, weight, symptom duration, or walking velocity (all *p* > 0.30). Sex distribution was also comparable between groups. In contrast, disease severity differed significantly between groups, with fallers exhibiting higher PDDS scores than nonfallers (*p* = 0.003, Cliff's *δ* = −0.55), indicating substantially greater self‐reported disability among individuals who experienced falls.

**TABLE 1:** Participant characteristics of the multiple sclerosis cohort stratified by six‐month fall status.

Variable	MS cohort (*n* = 43)	Nonfaller (*n* = 27)	Faller (*n* = 15)	*p*/Effect size
Age (years)	47.77 (9.98)	47.15 (9.94)	49.80 (9.82)	*p* = 0.411; *g* = −0.26
Height (m)	1.68 (0.11)	1.67 (0.10)	1.69 (0.11)	*p* = 0.480; *g* = −0.23
Weight (kg)	88.57 (23.26)	86.92 (23.43)	89.27 (22.70)	*p* = 0.753; *g* = −0.10
Symptom duration (years)	15.26 (9.64)	14.44 (9.97)	16.80 (9.50)	*p* = 0.455; *g* = −0.24
Forward walking Velocity (m/s)	0.98 (0.36)	1.03 (0.29)	0.89 (0.46)	*p* = 0.302; *g* = 0.38
Backward walking velocity (m/s)	0.69 (0.33)	0.73 (0.31)	0.61 (0.37)	*p* = 0.319; *g* = 0.34
Female, *n* (%)	34 (79.07)	23 (85.19)	10 (66.66)	—
Disease severity (PDDS)	1.0 (0–6)	1.0 (0–6)	4.0 (0–6)	*p* = 0.003; *δ* = −0.55
Prospective six‐month Falls	—	—	5.0 (0–60)	—

*Note:* Values are reported as mean (SD) unless otherwise indicated. Ordinal variables are reported as median values with ranges. Group differences (Nonfallers vs. Fallers) were assessed using Welch's *t*‐tests for continuous variables (effect sizes reported as Hedges' *g*), Wilcoxon rank‐sum tests for ordinal variables (effect sizes reported as Cliff's delta, *δ*). Ranges reflect minimum–maximum values. All *p* values are two‐tailed.

### 3.2 Regional Predictors of Forward and Backward Walking Speed

**FIGURE 1:**
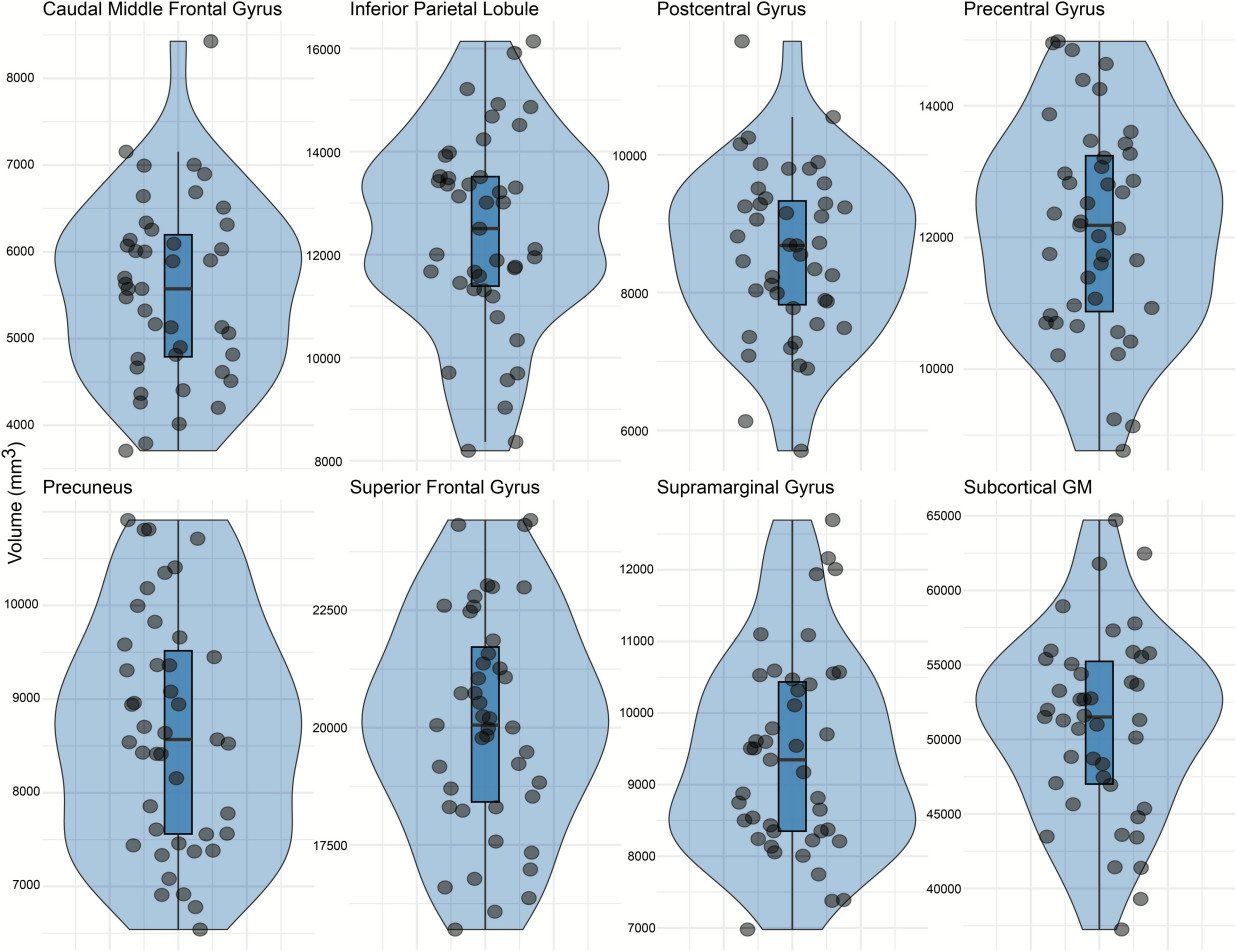
Distributions of regional cortical and subcortical volumes. Violin plots show the distribution of individual participant values (black dots) for each volumetric region of interest, including inferior parietal, postcentral, precentral, precuneus, superior frontal, subcortical gray matter, caudal middle frontal, and supramarginal cortices.

**FIGURE 2:**
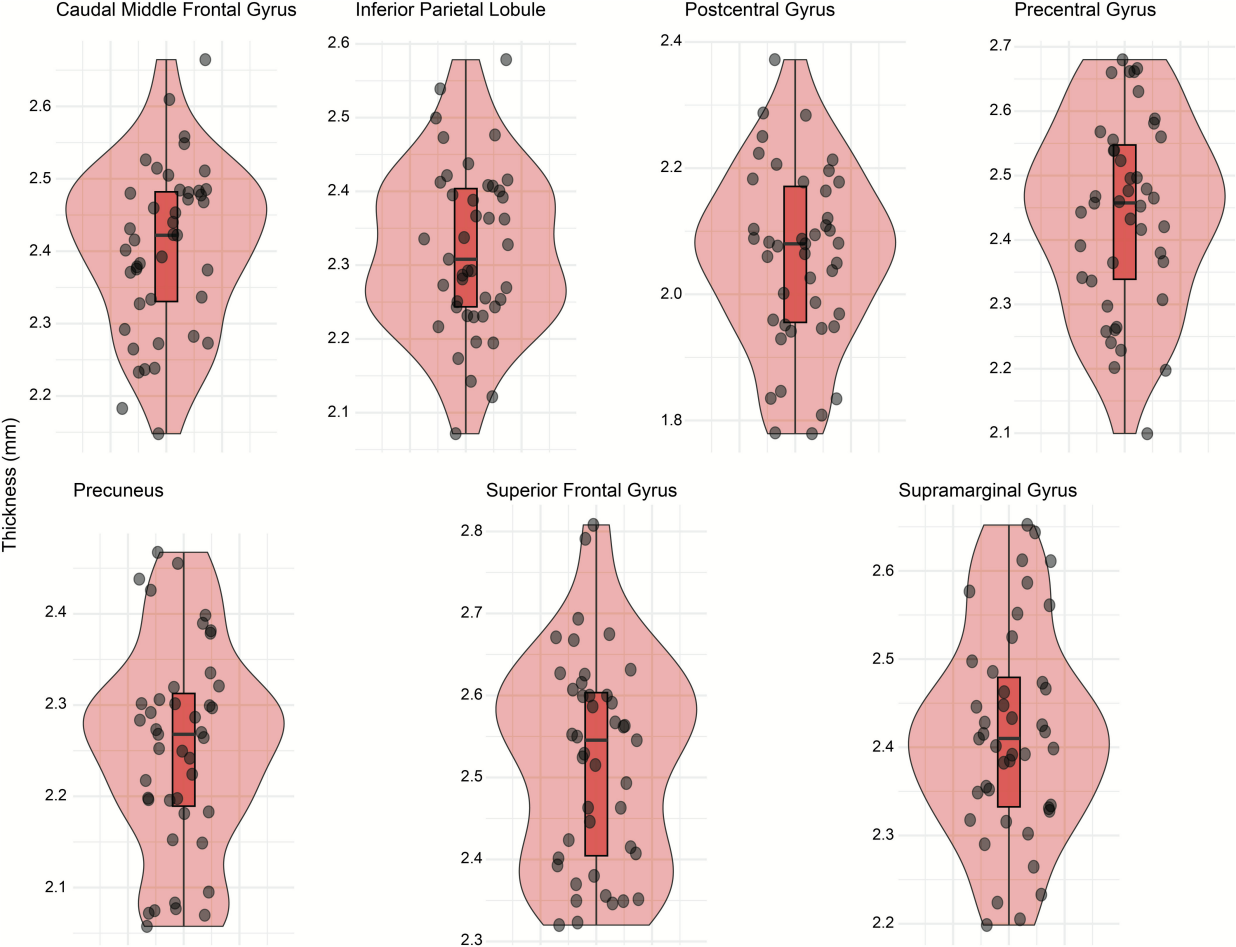
Distributions of regional cortical thickness. Violin plots display the distribution of individual participant values (black dots) for each cortical thickness region of interest, including inferior parietal, postcentral, precentral, precuneus, superior frontal, caudal middle frontal, and supramarginal cortices.

Distributions of regional cortical thickness and volume measures are shown in Figures 1 and 2.

#### 3.2.1 Volumetric ROIs

Volumetric models explained a small proportion of variance in forward (*F*(9, 33) = 1.56, *p* = 0.167, adjusted *R* ^2^ = 0.11) and backward (*F*(9, 33) = 1.73, *p* = 0.122, adjusted *R* ^2^ = 0.13) walking speeds. Associations for higher volumes and walking speed emerged for superior frontal (slower forward speeds) and caudal middle frontal and inferior parietal volumes (faster forward and backward speeds), but none survived FDR correction (Table S1).

#### 3.2.2 Cortical Thickness ROIs

For cortical thickness, the forward speed model did not reach significance (*F*(7, 35) = 2.01, *p* = 0.082, adjusted *R* ^2^ = 0.14), and no region achieved significance (all *p* > 0.26, *q* > 0.56). Backward speed was modestly predicted by cortical thickness (adjusted *R* ^2^ = 0.25), but no individual region was significant after correction; inferior parietal thickness showed only a trend (*β* = 2.18, *p* = 0.080, *q* = 0.321; Table S2).

**FIGURE 3:**
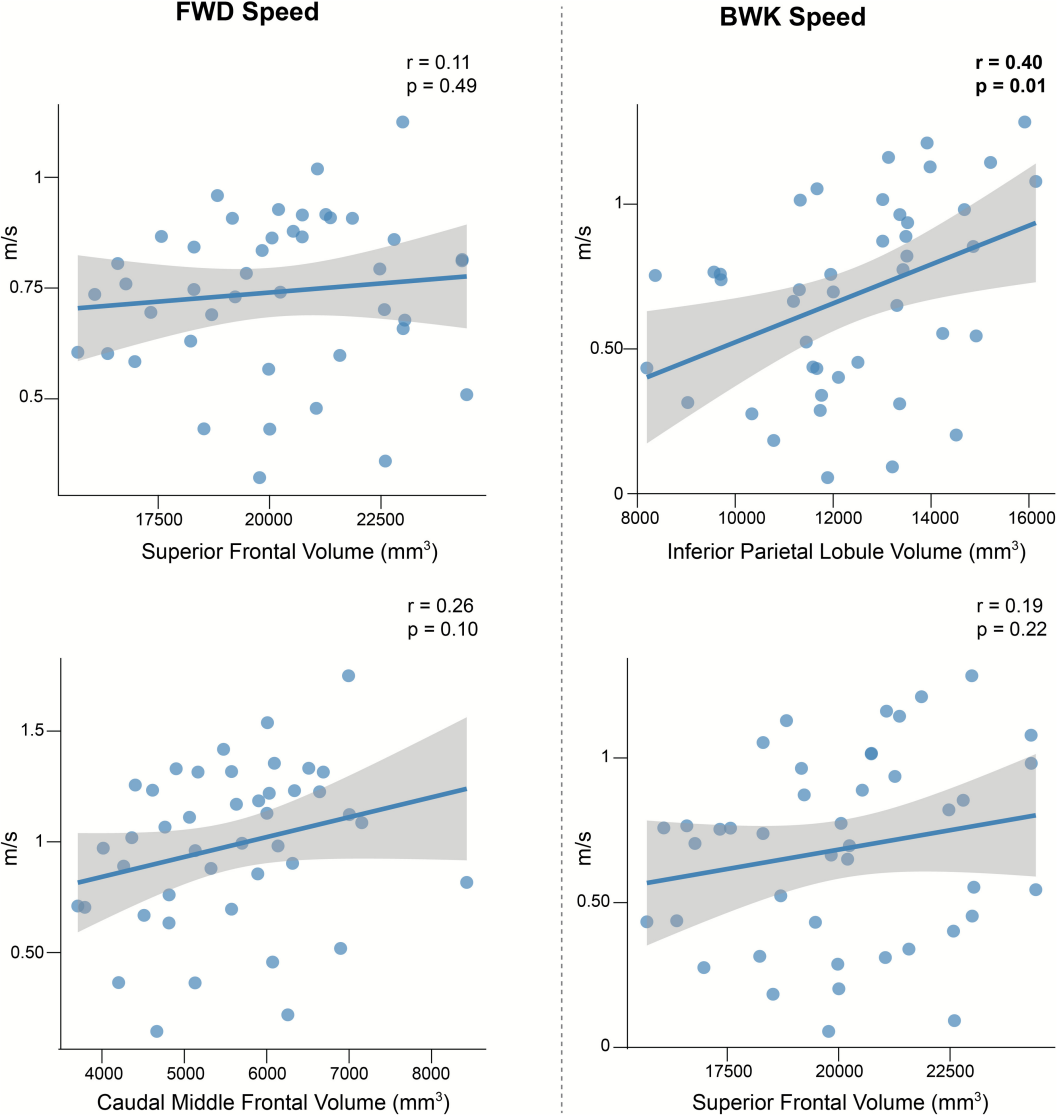
Regional associations between cortical structure and walking speed. Scatterplots show relationships between selected volumetric regions of interest and walking speed. Shaded areas represent 95% confidence intervals. Several regions, including inferior parietal, caudal middle frontal, and superior frontal cortices, demonstrated associations with gait outcomes; however, none survived false discovery rate correction. FWD = forward; BWK = backward.

In sum, regional models explained limited variance, and all effects disappeared after FDR correction. High collinearity among ROIs (VIFs 3–10) motivated the use of PCA to capture distributed patterns of atrophy (Figure 3).

### 3.3 Principal Component Analysis

**FIGURE 4:**
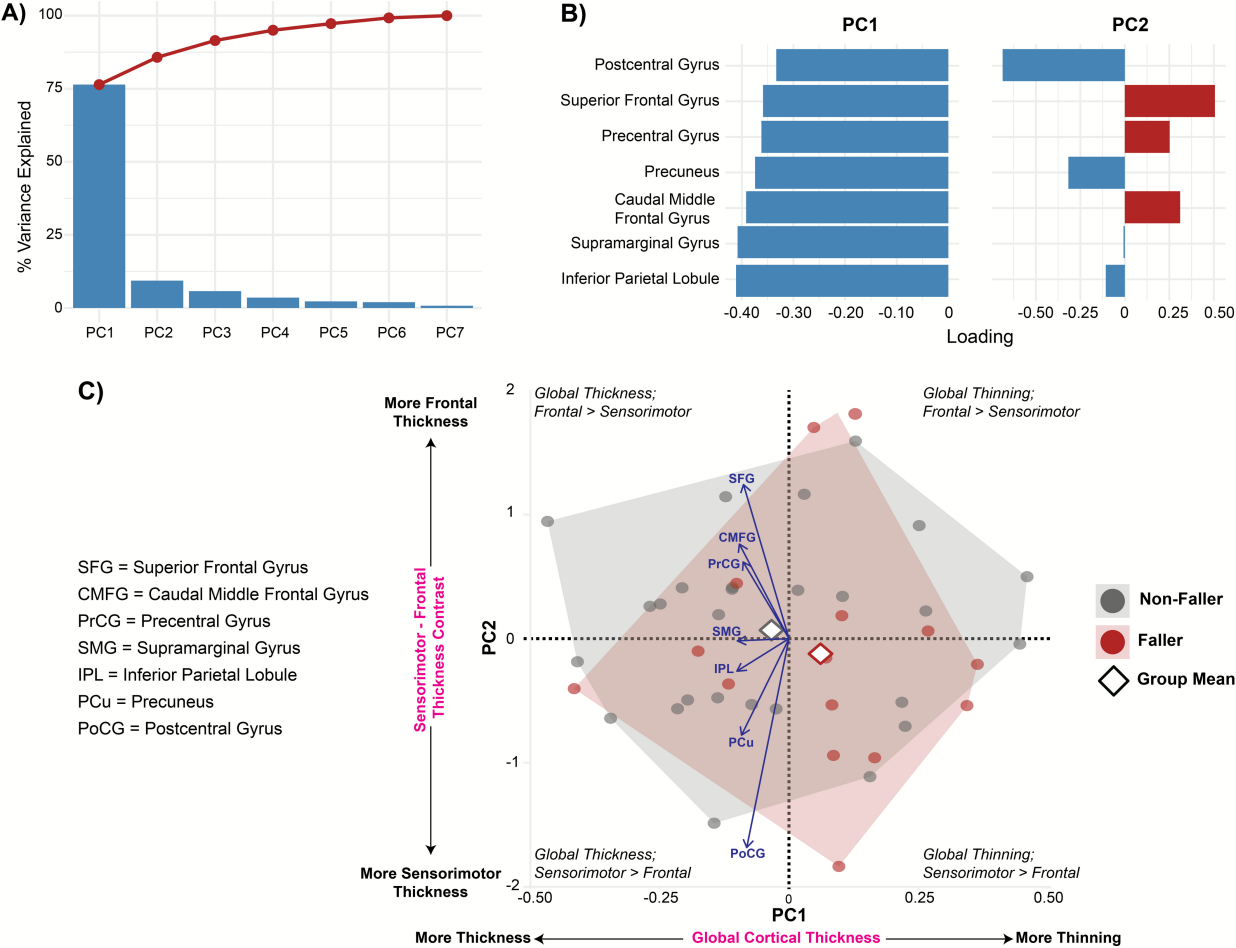
Cortical Thickness PCA Model. (A) Scree and cumulative variance plot. PC1 captures ~75% of total variance, PC2 ~ 10%, with remaining components each < 5%. (B) ROI loadings for PC1 (left) and PC2 (right). PC1 loads negatively and approximately equally on all regions, reflecting a global thinning factor, whereas PC2 contrasts frontal (positive loadings: superior frontal, caudal middle frontal) against sensorimotor cortices (negative loading: postcentral), indexing a frontal–sensorimotor thickness axis. (C) Biplot of individual PC1 × PC2 scores (*n*  =  42; gray  =  non‐fallers, red  =  fallers), with convex‐hull outlines and group‐mean diamonds. Arrow vectors show scaled ROI contributions. Along PC1, higher values reflect more cortical thinning (lower thickness), and more negative PC1 scores indicate greater overall thickness; along PC2, positive scores indicate relatively thicker frontal cortex compared to sensorimotor cortex. Fallers (red) tend toward lower PC1 (more thinning) and a modestly positive PC2 (frontal‐weighted profile).

Given the high inter‐regional collinearity (VIFs 3–10), PCA was performed separately on thickness and volume ROIs. The first thickness component (ThickPC1) loaded strongly and negatively on all regions (inferior parietal −0.41; supramarginal −0.41; caudal middle frontal −0.39; superior frontal −0.36), reflecting a global cortical thinning factor. ThickPC2 captured a sensorimotor–frontal contrast (postcentral: −0.72; superior frontal: 0.49). An overview of the cortical thickness PCA model is shown in Figure 4.

**FIGURE 5:**
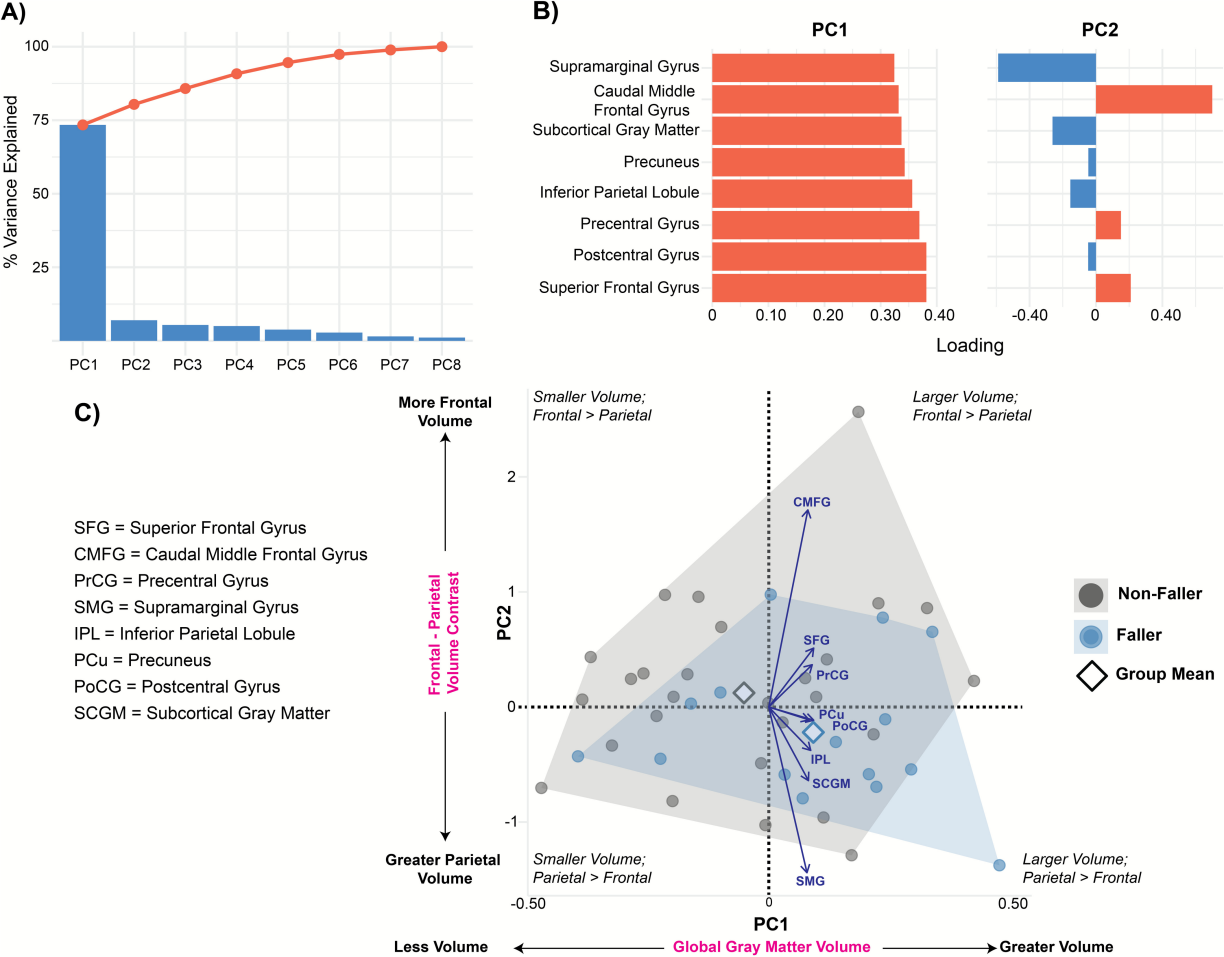
Cortical Volume PCA Model (A) Scree and cumulative variance plot. Volume PC1 captures ~78% of the total variance, PC2 ~ 9%, with all remaining PCs each < 4%. (B) ROI loadings for VolPC1 (left) and VolPC2 (right). VolPC1 loads positively and approximately equally on all eight regional volumes, including inferior parietal, postcentral, precentral, precuneus, superior frontal, caudal middle frontal, supramarginal, and subcortical gray matter, reflecting a global gray‐matter volume factor. VolPC2 contrasts frontal volumes (positive loadings: caudal middle frontal, precentral) against parietal volumes (negative loadings: supramarginal, inferior parietal), indexing a frontal–parietal volume axis. (C) Biplot of individual VolPC1 × VolPC2 scores (*n*  =  42 valid fall cases; gray  =  non‐fallers, blue  =  fallers), with convex‑hull outlines and group‑mean diamonds. Arrow vectors show scaled ROI contributions. Along VolPC1, higher scores indicate greater overall cortical gray–matter volume; lower scores indicate global atrophy. Along VolPC2, positive scores indicate a relatively frontal‑weighted volume profile, whereas negative scores indicate a relatively parietal‑weighted profile. Fallers (blue) tend toward lower VolPC1 (reduced global volume) and modestly higher VolPC2 (frontal‑weighted volume profile).

The volume PCA yielded a first component (VolPC1) that accounted for 78% of the total variance, with all eight regional volumes loading positively and nearly equally (inferior parietal = 0.356; postcentral = 0.381; precentral = 0.369; precuneus = 0.343; superior frontal = 0.381; caudal middle frontal = 0.332; supramarginal = 0.324; subcortical gray matter = 0.337), reflecting a global gray matter volume factor. The second component (VolPC2) explained 9% of variance and contrasted frontal and parietal regions: positive loadings on caudal middle frontal (0.700), superior frontal (0.209) and precentral (0.150) cortices versus negative loadings on supramarginal (−0.589), precuneus (−0.046) and inferior parietal (−0.154), indexing a frontal–parietal volume dissociation. An overview of the cortical/subcortical volumetric PCA model is shown in Figure 5.

### 3.4 PCA‐Derived Predictors of Forward and Backward Walking Speed

**FIGURE 6:**
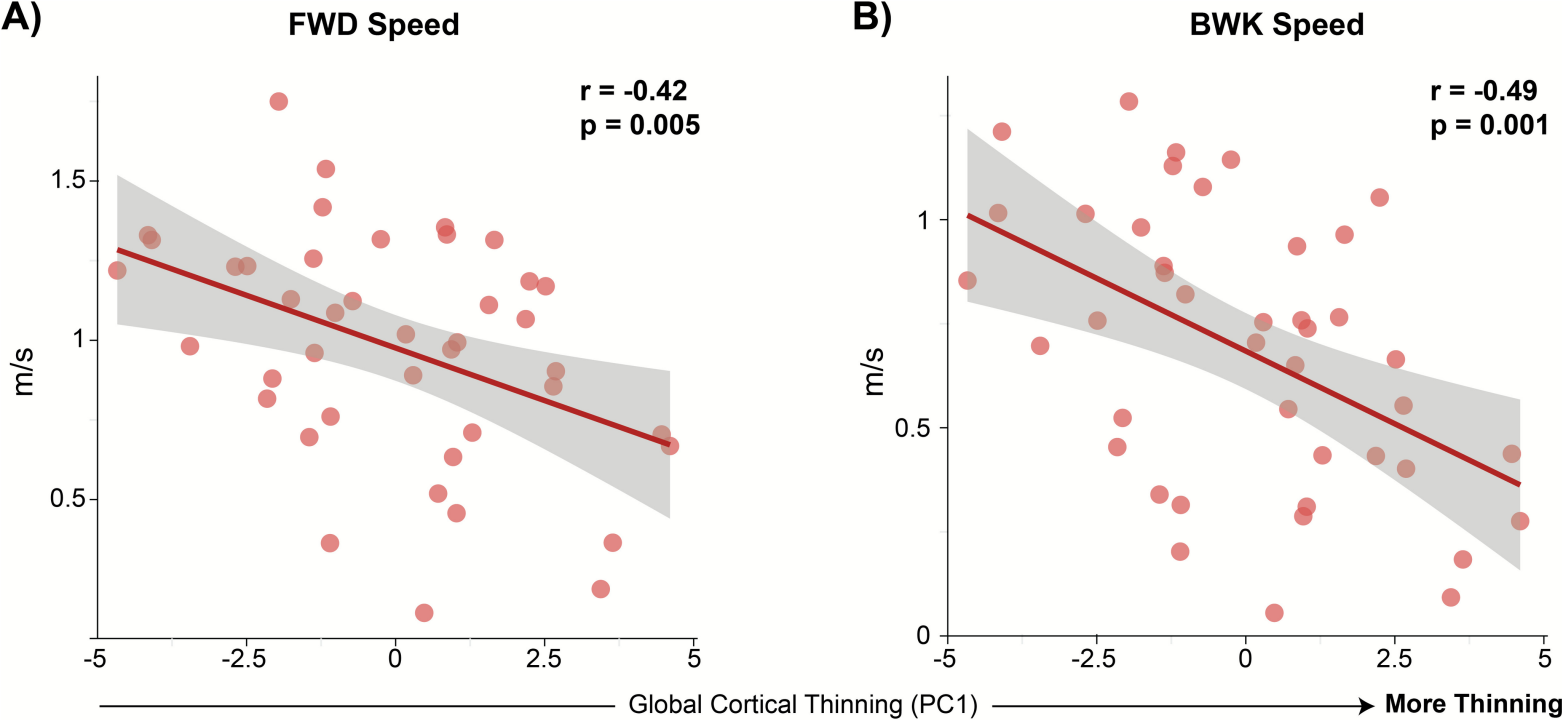
Associations between global cortical thinning (ThickPC1) and walking speed. Each panel shows individual participants and 95% confidence band (shaded gray). Higher ThickPC1 scores reflect more global cortical thinning (lower overall thickness). FWD = forward; BWK = backward.

Global cortical thinning (Thick PC1) was the only PC that reliably predicted gait speeds. Specifically, greater thinning (greater Thick PC1 scores) was associated with slower forward walking speed (*β* = −0.065, SE = 0.025, 95% CI [−0.115, −0.015], *p* = 0.0137, *q* = 0.055; Adjusted *R* ^2^ = 0.14) and slower backward walking speed (*β* = −0.061, SE = 0.022, 95% CI [−0.105, −0.017], *p* = 0.0092, *q* = 0.037; Adjusted *R* ^2^ = 0.19). Cross‐validation indicated that these associations generalized beyond the sample, with CV *R* ^2^ values of 0.32 (forward) and 0.40 (backward), though prediction errors remained moderate (CV RMSE 0.35 and 0.33 m/s, respectively). Neither the frontal‐sensorimotor contrast (Thick PC2) nor either volume component (Vol PC1, Vol PC2) predicted gait speed after correction (all *q* > 0.58; Table 2, Figure 6).

**TABLE 2:** PCA predictors of forward and backward walking speed.

Predictor	Estimate (*β*)	SE	95% CI	*p*	*q*‐value
Forward walking speed (m/s)					
ThickPC1	−0.065	0.025	[−0.115, −0.015]	**0.014**	0.055
VolPC1	0.003	0.026	[−0.048, 0.054]	0.914	0.980
ThickPC2	−0.077	0.072	[−0.218, 0.064]	0.291	0.582
VolPC2	−0.0012	0.070	[−0.140, 0.137]	0.980	0.980
Backward walking speed (m/s)					
ThickPC1	−0.061	0.022	[−0.105, −0.017]	**0.010**	**0.037**
VolPC1	0.021	0.023	[−0.024, 0.067]	0.359	0.718
ThickPC2	−0.024	0.064	[−0.149, 0.102]	0.711	0.948
VolPC2	−0.020	0.062	[−0.142, 0.102]	0.752	0.948

*Note:* Unstandardized *β* coefficients represent the change in gait speed (m/s) per 1‐unit increase in each principal component. SE = standard error; *q*‐values are Benjamini–Hochberg FDR‐adjusted across the four PCA predictors per model. Thick PC1 reflects global cortical thinning (all ROIs load negatively): higher scores correspond to greater thinning. Thick PC2 captures a frontal > sensorimotor thickness contrast (frontal gyri load positively, postcentral negatively), so higher scores indicate relatively thicker frontal versus sensorimotor cortex. Vol PC1 represents global gray matter volume (all ROIs load positively): higher scores indicate greater total gray matter volume. Vol PC2 represents a frontal > parietal volume contrast (caudal middle frontal loads positively, supramarginal negatively), with higher scores reflecting relatively larger frontal than parietal volumes. Bolded values indicate predictors showing statistically significant (*q* < 0.05) with gait speed after false discovery rate correction.

### 3.5 Group Comparisons and Moderation of Gait and Cortical Thinning by Six‐Month Prospective Falls

**FIGURE 7:**
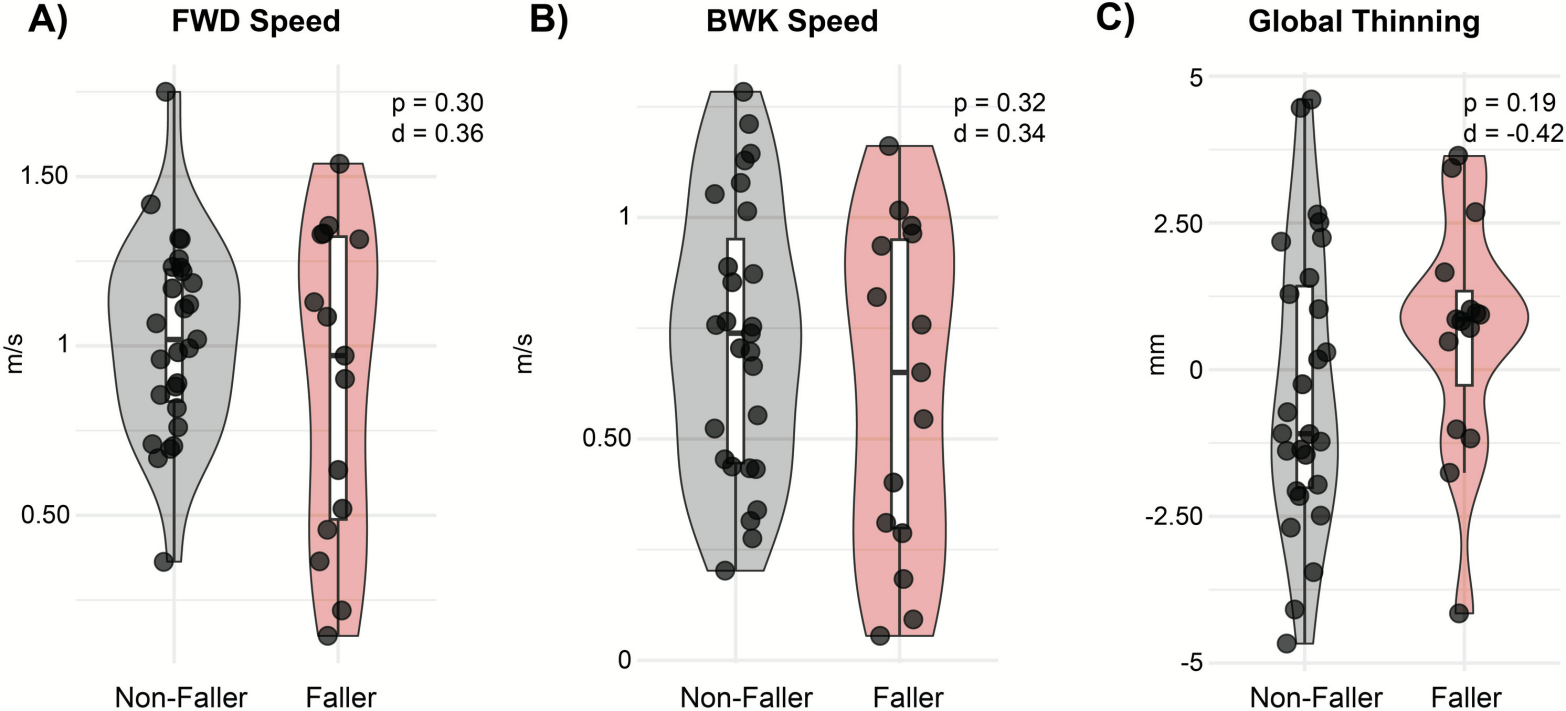
Group comparisons of gait and cortical thinning between fallers and nonfallers. Violin plots display individual participant values (black dots) and distribution density for forward (FWD) comfortable speed (A), backward (BWK) comfortable speed (B), global cortical thinning (ThickPC1; C). Fallers are shown in red and nonfallers in gray. No significant group differences were observed on any measure.

Fallers and nonfallers did not differ in walking speed or global cortical thinning (Figure **7**). No group differences were observed for forward (*t* (20.2) = 1.06, *p* = 0.302, *d* = 0.36) or backward (*t* (24.9) = 1.02, *p* = 0.319, *d* = 0.34) walking speed. Likewise, global cortical thinning (ThickPC1) was comparable across groups (*t* (33.8) = −1.35, *p* = 0.185, *d* = −0.42).

**FIGURE 8:**
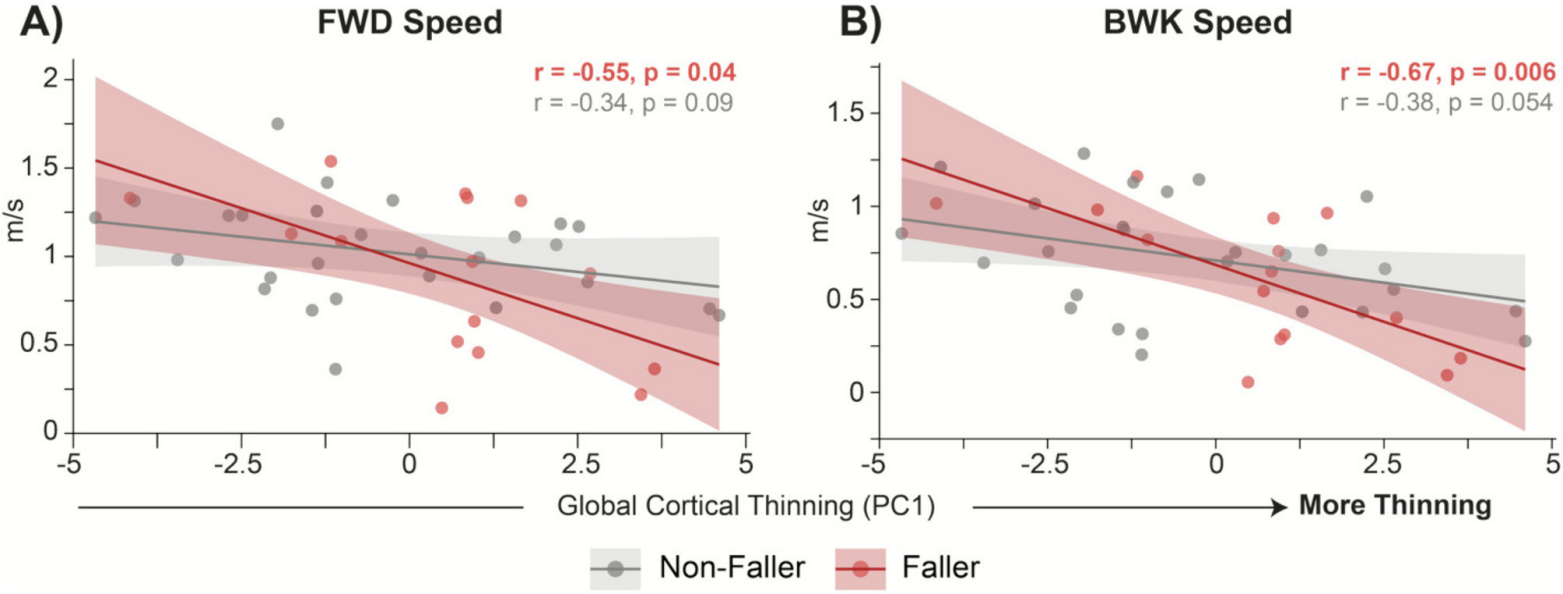
The relationship between global cortical thinning (ThickPC1) and walking speed by fall status. Scatterplots illustrate associations between global cortical thinning (ThickPC1) and walking speed in fallers (red) and non‐fallers (gray). Fallers showed stronger negative associations between cortical thinning and gait speed compared to non‐fallers, although these moderation effects did not remain significant after FDR correction (see Table 3 for model statistics). FWD = forward; BWK = backward.

Fall status did not significantly moderate the association between global cortical thinning (ThickPC1) and walking speed after FDR adjustment. For forward walking speed, the interaction term was negative but nonsignificant (*β* = −0.085, SE = 0.050, Wald 95% CI [−0.183, 0.014], raw *p* = 0.100, *q* = 0.207). A similar negative trend was observed for backward walking speed (*β* = −0.075, SE = 0.045, Wald 95% CI [−0.162, 0.013], raw *p* = 0.104, *q* = 0.207). Complementary bootstrap analyses yielded 95% CIs that narrowly excluded zero (forward: −0.174 to −0.005; backward: −0.162 to −0.017), suggesting a potential negative moderation effect that did not survive multiple‐comparison correction. These moderation patterns are illustrated in Figure 8, with full model estimates provided in Table 3.

**TABLE 3:** Moderation of global thinning by fall status on comfortable walking speeds.

Predictor	Estimate (*β*)	SE	95% CI	*p*‐value	*q*‐value
Forward walking speed (m/s)					
ThickPC1	−0.040	0.026	[−0.091, 0.012]	0.138	0.207
Fall status	−0.050	0.108	[−0.262, 0.162]	0.647	0.776
ThickPC1 × fall status	−0.085	0.050	[−0.183, 0.014]	0.100	0.207
Backward walking speed (m/s)					
ThickPC1	−0.048	0.023	[−0.093, −0.002]	0.048	0.207
Fall status	−0.024	0.096	[−0.212, 0.164]	0.807	0.807
ThickPC1 × fall status	−0.075	0.045	[−0.162, 0.013]	0.104	0.207

*Note:* Unstandardized *β* coefficients represent the change in walking speed (m/s) per 1‐unit increase in each predictor. SE = standard error of the estimate; *q*‐values are Benjamini–Hochberg FDR‐adjusted across the four interaction tests. ThickPC1 reflects global cortical thinning (higher scores = greater thinning).

## 4 Discussion

This study aimed to characterize how patterns of gray‐matter morphometry underpin walking capacity and prospective falls in MS. We found that a global cortical thinning signature, capturing diffuse reductions in sensorimotor and frontal thickness, was the most consistent correlate of slower gait speeds, and that this association, despite the interaction bordering on significance, was particularly pronounced in individuals with recent falls. These results suggest that widespread cortical atrophy, rather than isolated regional changes, critically undermines mobility and exacerbates fall vulnerability in MS. Collectively, our findings nominate global cortical thickness as a potential biomarker of gait impairment and fall risk, with clear implications for monitoring disease progression and guiding targeted rehabilitation strategies.

Beyond individual ROIs, our multivariate analyses demonstrated that a global cortical thinning emerged as a consistent correlate of gait slowing. While nominal associations were observed in specific regions (e.g., superior frontal, caudal middle frontal, and inferior parietal; Figure 3), none survived FDR correction, emphasizing the limitations of univariate, region‐by‐region approaches in small samples (Swanson and Fling 2023; Geisseler et al. 2016). Instead, PCA captured distributed patterns of atrophy, revealing that diffuse cortical thinning best explained gait slowing (Table 2, Figure 6). Although both cortical thickness and gray‐matter volume were evaluated, only cortical thinning emerged as a consistent correlate of gait slowing, suggesting that thickness‐based measures may be more sensitive to mobility‐related neurodegeneration in this cohort. This aligns with emerging evidence suggesting that global cortical atrophy is more closely linked to motor function and disability in MS than isolated cortical changes (Sailer et al. 2003; Pareto et al. 2015; Tillema et al. 2016).

Backward walking speed showed numerically stronger associations with global cortical thinning than forward walking, although the difference in explained variance between conditions was modest. This trending distinction is consistent with prior work indicating that backward gait requires greater sensorimotor integration, cognitive demand, and postural control, and is disproportionately affected in MS (Takla et al. 2023; Monaghan, VanNostrand, and Fritz 2024). Recent myelin water imaging data extend this view, showing that backward walking velocity is linked to cerebellar peduncle integrity, whereas forward walking speed is tied to callosal pathways (Monaghan et al. 2025). Because the cerebellar peduncles are critical for integrating proprioceptive and vestibular feedback with higher‐order control, these findings reinforce that backward walking places unique demands on both sensory and cognitive systems. Together, this evidence suggests that backward walking velocity may serve as a more sensitive behavioral index of cortical and cerebellar structural degeneration in MS than traditional forward walking measures.

Although we hypothesized that individuals with recent falls would exhibit greater cortical thinning and slower gait, no significant group differences emerged in either structural or gait metrics (Figure 7). This is consistent with recent findings showing that fallers are a highly heterogeneous group, in which compensatory mechanisms, cognitive reserve, or variability in MS subtype can obscure group‐level effects (Coote et al. 2020; Edwards et al. 2023; Kaddoura et al. 2024). However, exploratory moderation analyses suggested that the association between cortical thinning and gait speed may be greater among fallers, even though these interactions narrowly missed significance after correction. In particular, the effect sizes for thinning‐gait correlations were larger among fallers, pointing to a potentially latent vulnerability profile that may not be detectable in binary group comparisons. This pattern, observed in a functionally high‐performing cohort (as reflected by low PDDS scores), underscores the need for larger, more heterogeneous samples to clarify these interactions. The absence of group differences also highlights the multifactorial nature of falls in MS, indicating that factors beyond walking speed, such as balance control, sensory integration, cognition, and environmental context, likely contribute to fall risk.

Backward walking emerged as a potentially informative behavioral measure. Previous studies have shown that backward walking velocity not only correlates with MS‐related cognitive and sensorimotor deficits but also outperforms forward walking in predicting fall status (Edwards et al. 2020). Moreover, pilot data from an eight‐week backward walking training case series suggest that backward walking training may induce both structural and functional adaptations, improving gait and balance and possibly white matter integrity in cerebellar and callosal pathways, further underscoring its relevance for tracking and potentially mitigating fall risk (Abbawi et al. 2025). Taken with our findings, this suggests that backward walking, combined with neuroanatomical measures such as global cortical thickness, may help identify and perhaps even ameliorate fall risk in early or high‐functioning stages of MS.

Several limitations should be noted. The modest sample size limited power for detecting small‐to‐moderate effects and reduced the reliability of interaction estimates. We attempted to address this through dimensionality reduction (PCA), correction for multiple comparisons (FDR), and 10‐fold cross‐validation. Given the modest sample size, we additionally provide effect‐size–based sample size estimates derived from the primary global cortical thinning‐gait associations to inform future study planning (Supporting Information). These estimates suggest that approximately 44–49 participants would be required for 80% power and 57–64 participants for 90% power to detect associations of similar magnitude; however, because effect sizes from modest samples may be upwardly biased, larger samples may be required for stable estimation. Relatedly, although subcortical gray matter was included as a total volume measure, individual subcortical regions, as well as cerebellar and brainstem structures, were not examined. These regions are likely important for gait and falls but were beyond the scope of the current study due to power limitations. Future research with larger samples and lesion‐aware pipelines should explicitly investigate these structures. The prospective fall data lend ecological validity, but our sample's relatively low disability (PDDS scores) may have attenuated the detection of structural or motor differences between fallers and nonfallers, and limited generalizability to persons with MS with greater disease burden. Moreover, while morphometric measures were carefully quality‐controlled, lesion burden and lesion‐filling procedures were not modeled, which could introduce bias in cortical thickness estimates. Future work should integrate lesion mapping with multimodal imaging approaches that integrate lesion‐aware morphometry with diffusion and functional measures, alongside more ecological assessments of gait using wearable sensors and real‐world mobility tasks, to better capture the distributed neural systems supporting locomotion in MS.

## 5 Conclusion

This study provides preliminary evidence that cortical thinning is associated with slower forward and backward walking speed in people with MS. Although backward walking displayed numerically stronger associations, these effects were modest; further well‐powered studies are necessary to determine their specificity and clinical significance. Although differences in structure and gait by fall status were not statistically significant, the stronger thinning–gait associations observed in fallers point to a potential biomarker of fall vulnerability. Our findings suggest that backward walking velocity may serve as a measure to detect early mobility decline in MS. However, given the modest sample size, these results should be interpreted cautiously. Larger, adequately powered studies are needed to confirm these effects, formally test interactions with fall status, and determine whether cortical thinning prospectively predicts fall events. Future research should employ longitudinal designs, multimodal neuroimaging, and more ecologically valid gait assessments to establish whether these structural markers can be integrated into fall risk prediction models and inform individualized rehabilitation strategies.

## Author Contributions

**A. S. Monaghan:** conceptualization, formal analysis, methodology, project administration, visualization, writing – original draft. **P. G. Monaghan:** conceptualization, data curation, formal analysis, methodology, writing – original draft. **T. N. Takla:** data curation, investigation, project administration, writing – review and editing. **N. E. Fritz:** conceptualization, funding acquisition, investigation, methodology, project administration, resources, supervision, writing – review and editing.

## Funding

This study was supported by Clinical Center National Institutes of Health grants (F31HD116491, R21HD106133) and was also supported by National Multiple Sclerosis Society grants (MB–2107–38295).

## Ethics Statement

All procedures performed in this study were in accordance with the ethical standards of the institutional and/or national research committee and with the 1964 Helsinki Declaration and its later amendments or comparable ethical standards. The study was approved by the Institutional Review Board at Wayne State University.

## Consent

All participants completed written informed consent prior to study participation.

## Conflicts of Interest

The authors declare no conflicts of interest.

## Supporting information


**Table S1:** Cortical volume predictors of forward and backward walking speed.
**Table S2:** Cortical thickness predictors of forward and backward walking speed.

## Data Availability

Data are available upon reasonable request.
