# Supplementary material for: Global Cortical Thinning Predicts Slower Forward and Backward Walking in Multiple Sclerosis
Source: Eur J Neurosci. 2026 Jan 21;63(2):e70412. doi: 10.1111/ejn.70412 (PMC12823345; doi:10.1111/ejn.70412)
Supplement: Supplementary file 1 — Table S1: Cortical volume predictors of forward and backward walking speed. Table S2: Cortical thickness predictors of forward and backward walking speed. [file EJN-63-0-s001.pdf]

Supplemental Table 1. Cortical Volume Predictors of Forward and Backward Walking Speed

Predictor	Estimate (β)	SE	95% CI	p-value	q-value
Forward Walking Speed					
Inferior parietal	1.00E-04	5.17E-05	[-4.98e-06, 2.05e-04]	0.061	0.204
Postcentral	7.91E-05	1.09E-04	[-1.42e-04, 3.00e-04]	0.473	0.638
Precentral	-6.79E-05	8.18E-05	[-2.34e-04, 9.85e-05]	0.413	0.638
Precuneus	-5.46E-05	8.19E-05	[-2.21e-04, 1.12e-04]	0.510	0.638
Superior frontal	-1.20E-04	5.39E-05	[-2.29e-04, -1.03e-06]	0.033	0.204
Subcortical GM	2.27E-05	1.44E-05	[-6.56e-06, 5.19e-05]	0.124	0.310
Caudal middle frontal	1.90E-04	9.23E-05	[2.32e-06, 3.78e-04]	0.047	0.204
Supramarginal	-8.32E-06	5.77E-05	[-1.26e-04, 1.09e-04]	0.886	0.886
Estimated intracranial	-9.45E-08	3.74E-07	[-8.56e-07, 6.67e-07]	0.802	0.886
Backward Walking Speed					
Inferior parietal	1.05E-04	4.65E-05	[9.95e-06, 1.99e-04]	0.031	0.169
Postcentral	8.07E-05	9.80E-05	[-1.19e-04, 2.80e-04]	0.416	0.705
Precentral	-5.19E-05	7.37E-05	[-2.02e-04, 9.80e-05]	0.486	0.705
Precuneus	-4.30E-05	7.38E-05	[-1.93e-04, 1.07e-04]	0.564	0.705
Superior frontal	-1.07E-04	4.85E-05	[-2.06e-04, -8.72e-06]	0.034	0.169
Subcortical GM	1.51E-05	1.29E-05	[-1.13e-05, 4.14e-05]	0.253	0.632
Caudal middle frontal	1.54E-04	8.32E-05	[-1.57e-05, 3.23e-04]	0.074	0.246
Supramarginal	3.97E-06	5.20E-05	[-1.02e-04, 1.10e-04]	0.940	0.940
Estimated intracranial	-6.53E-08	3.37E-07	[-7.51e-07, 6.21e-07]	0.848	0.940

Unstandardized β coefficients represent the change in walking speed (m/s) per 1 mm^3 increase in regional volume. Volumes (including subcortical gray matter and estimated total intracranial volume) are expressed in cubic millimeters. SE = standard error of the estimate; 95 % CI = bias-corrected and accelerated bootstrap confidence interval (1,000 resamples). p-values derive from each predictor's t-test. q-values are Benjamini–Hochberg false discovery rate–adjusted p-values correcting for nine simultaneous volume comparisons per model. All models include only volumetric predictors (no additional covariates).

Supplemental Table 2. Cortical Thickness Predictors of Forward and Backward Walking Speed

Predictor	Estimate (β)	SE	95% CI	p-value	q-value
Forward Walking Speed					
Inferior parietal	1.367	1.410	[-1.50, 4.23]	0.339	0.897
Postcentral	0.061	0.642	[-1.24, 1.37]	0.925	0.925
Precentral	0.386	0.658	[-0.95, 1.72]	0.561	0.897
Precuneus	0.245	0.980	[-1.74, 2.23]	0.804	0.919
Superior frontal	-0.236	0.906	[-2.07, 1.60]	0.796	0.919
Caudal middle frontal	-1.273	1.130	[-3.57, 1.02]	0.268	0.897
Supramarginal	0.804	1.362	[-1.96, 3.57]	0.559	0.897
Backward Walking Speed					
Inferior parietal	2.179	1.209	[-0.28, 4.63]	0.080	0.321
Postcentral	-0.310	0.551	[-1.43, 0.81]	0.578	0.726
Precentral	0.270	0.564	[-0.87, 1.41]	0.635	0.726
Precuneus	-0.052	0.840	[-1.76, 1.65]	0.951	0.951
Superior frontal	-0.439	0.777	[-2.02, 1.14]	0.576	0.726
Caudal middle frontal	-1.106	0.969	[-3.07, 0.86]	0.261	0.697
Supramarginal	0.825	1.168	[-1.55, 3.20]	0.485	0.726

Unstandardized β coefficients represent the change in walking speed (m/s) per 1 mm increase in regional cortical thickness. Thickness measures are expressed in millimeters. SE = standard error of the estimate; 95 % CI = bias-corrected and accelerated bootstrap confidence interval (1,000 resamples). p-values derive from each predictor's t-test. q-values are Benjamini–Hochberg false discovery rate–adjusted p-values correcting for seven simultaneous thickness comparisons per model. All models include only thickness predictors (no additional covariates).

Supplemental Methods: Effect-Size-Based Sample Size Estimation

To aid study planning and provide context for the modest sample size of this study, we calculated effect-size-based sample size estimates for the primary outcomes using observed links between global cortical thinning and gait performance. These estimates are meant for planning future research, not for retrospective significance interpretation.

Derivation of Partial Correlation Coefficients

Effect sizes were obtained by converting the *t*-statistics associated with the global cortical thinning component (ThickPC1) in the primary regression models (Table 2) into partial correlation coefficients (*r*). This approach follows the standard relationship between the *t*-statistic, residual degrees of freedom (*df*), and the partial correlation:

$$r = \sqrt{\frac{t^2}{t^2 + df}}$$

From Table 2, the regression coefficients for ThickPC1 were:

- **Forward walking speed:** β = -0.065, SE = 0.025

$$t = \frac{-0.065}{0.025} = -2.60$$

- **Backward walking speed:** β = -0.061, SE = 0.022

$$t = \frac{-0.061}{0.022} \approx -2.77$$

The principal component gait models included four predictors (ThickPC1, ThickPC2, VolPC1, VolPC2). With *n* = 42 participants contributing valid data to these analyses, the residual degrees of freedom were:

$$df = n - (p + 1) = 42 - (4 + 1) = 37$$

Substituting these values into the equation above yielded the following partial correlations:

- **Forward walking:**

$$r = \sqrt{\frac{2.60^2}{2.60^2 + 37}} = \sqrt{\frac{6.76}{43.76}} = 0.392 \approx 0.39$$

- **Backward walking:**

$$r = \sqrt{\frac{2.77^2}{2.77^2 + 37}} = \sqrt{\frac{7.69}{44.69}} = 0.414 \approx 0.41$$

These values represent the partial associations between global cortical thinning and walking speed, controlling for the other PCA-derived structural components in the model.

Sample Size Estimation

Using these partial correlation coefficients, sample size estimates were computed using Fisher's *z*-transformation for correlation power (two-tailed $\alpha = 0.05$). Estimated sample sizes required to detect associations of similar magnitude were approximately:

- **Forward walking:**
  ~49 participants for 80% power; ~64 participants for 90% power
- **Backward walking:**
  ~44 participants for 80% power; ~57 participants for 90% power

Because effect sizes derived from modest samples may be upwardly biased, these estimates should be interpreted conservatively. Future studies may wish to plan for smaller effects (i.e., larger samples) to ensure stable estimation and adequate power.
